# Identification of Potential Prophylactic Medical Countermeasures Against Acute Radiation Syndrome (ARS)

**DOI:** 10.3390/ijms26094055

**Published:** 2025-04-25

**Authors:** Kia T. Liermann-Wooldrik, Arpita Chatterjee, Elizabeth A. Kosmacek, Molly S. Myers, Oluwaseun Adebisi, Louise Monga-Wells, Liu Mei, Michelle P. Takacs, Patrick H. Dussault, Daniel R. Draney, Robert Powers, James W. Checco, Chittibabu Guda, Tomáš Helikar, David B. Berkowitz, Kenneth W. Bayles, Alan H. Epstein, Lynnette Cary, Daryl J. Murry, Rebecca E. Oberley-Deegan

**Affiliations:** 1 Department of Biochemistry and Molecular Biology, University of Nebraska Medical Center, Omaha, NE 68198, USAarpita.chatterjee@unmc.edu (A.C.); louise.monga@unmc.edu (L.M.-W.);; 2 Department of Chemistry, University of Nebraska-Lincoln, Lincoln, NE 68588, USApatrick.dussault@unl.edu (P.H.D.); checco@unl.edu (J.W.C.); dberkowitz1@unl.edu (D.B.B.); 3 Nebraska Center for Integrated Biomolecular Communication, University of Nebraska-Lincoln, Lincoln, NE 68588, USA; 4 Department of Genetics, Cell Biology and Anatomy, University of Nebraska Medical Center, Omaha, NE 68198, USA; babu.guda@unmc.edu; 5 Center for Biomedical Informatics Research and Innovation, University of Nebraska Medical Center, Omaha, NE 68198, USA; 6 Department of Biochemistry, University of Nebraska-Lincoln, Lincoln, NE 68588, USA; 7 Department of Pathology, Microbiology, and Immunology, University of Nebraska Medical Center, Omaha, NE 68198, USA; 8 Occupational and Environmental Medicine, Defense Health Agency, Falls Church, VA 22042, USA; alan.h.epstein.civ@health.mil; 9 Division of Radioprotectants, Department of Pharmacology and Molecular Therapeutics, F. Edward Hébert School of Medicine, Uniformed Services University of the Health Sciences, Bethesda, MD 20814, USA; 10 Armed Forces Radiobiology Research Institute, Uniformed Services University of the Health Sciences, Bethesda, MD 20814, USA; 11 Department of Pharmacy Practice and Science, University of Nebraska Medical Center, Omaha, NE 68198, USA

**Keywords:** acute radiation syndrome, radioprotection, DNA damage

## Abstract

Acute radiation syndrome (ARS) occurs when hematopoietic or gastrointestinal cells are damaged by radiation exposure causing DNA damage to the bone marrow and gastrointestinal epithelial stem cell populations. In these highly proliferative cell types, DNA damage inhibits stem cell repopulation. In humans and animals, this inability to regenerate stem cells is lethal. Within this manuscript, several compounds, Amifostine, Captopril, Ciprofloxacin, PrC-210, 5-AED (5-androstene-3β,17β-diol), and 5-AET (5-androstene-3β,7β,17B-triol), are assessed for their ability to protect against ARS in an in vitro and/or in vivo setting. ARS was accomplished by irradiating mouse bone marrow cells or rat intestinal epithelial (IEC-6) cells in vitro with 4–8 Gy and in vivo by exposing *Mus musculus* to 7.3 Gy of whole-body irradiation. The primary endpoints of this study include cellular viability, DNA damage via γ-H2AX, colony formation, and overall survival at 30-days post-irradiation. In addition to evaluating the radioprotective performance of each compound, this study establishes a distinct set of in vitro assays to predict the overall efficacy of potential radioprotectors in an in vivo model of ARS. Furthermore, these results highlight the need for FDA-approved medical intervention to protect against ARS.

## 1. Introduction

Radiation damages cells by two well-described mechanisms. The first is direct damage that results from high-energy particles causing DNA double-strand breaks [1]. The second is indirect damage from reactive oxygen species (ROS), which are present seconds to days after radiation exposure. ROS damages DNA, RNA, proteins, and lipids, ultimately resulting in cell death [1,2,3,4,5].

Exposure to whole-body high-dose radiation can manifest in acute radiation syndrome (ARS) due to damage to the hematopoietic system and the gastrointestinal (GI) tract [1]. Blood cells and GI cells are especially prone to radiation damage because they are highly proliferative, making their DNA more vulnerable to damage [6,7]. The bone marrow continuously produces new blood cells to replace the old and damaged ones [7]. The epithelium of the GI tract is completely replaced every 3–4 days. Both cellular compartments rely on progenitor stem cells to repopulate their tissues. Accordingly, if the stem cells are damaged, the bone marrow or GI tract cannot regenerate, and this results in the death of the animal [6]. Thus, to protect from ARS, the bone marrow and GI stem cell populations need to remain viable.

Radioprotectors are drugs that are administered prophylactically to reduce radiation damage. There have been many studies in the past 40 years that have identified some promising candidates as radioprotectors [8]. These drugs typically scavenge ROS, enhance DNA repair, boost stem cell production, or inhibit cell cycling during radiation exposure. The only FDA-approved radioprotector is Amifostine, and it is only approved for use in cancer patients [9]. Amifostine is efficacious as a radioprotector, due to its potent ROS scavenging capabilities, but causes such severe side effects that it is not commonly used [10]. There are currently no FDA-approved drugs to be used as ARS radioprotectors [8]. The reasons for this are that ARS radioprotection is not a lucrative market, and it is expensive to conduct all the testing required for approval from the FDA. However, military personnel who must enter a nuclear blast site, first responders exposed to a dirty bomb, or workers who need to clean up a nuclear plant meltdown would benefit greatly from the development of radioprotectors.

The goal of this manuscript is to compare the radioprotectors identified in the literature and determine which compounds to pursue as viable radioprotectors moving forward in a non-biased fashion. Five drugs were selected from the literature based on efficacy, safety, and drug readiness for use as a radioprotector from ARS: Ciprofloxacin, Captopril, PrC-210, 5-AED (5-androstene-3β,17β-diol), and 5-AET (5-androstene-3β,7β,17B-triol).

Ciprofloxacin is an FDA-approved fluoroquinolone antibiotic that has antimicrobial activity against Gram-positive and Gram-negative bacteria and has a good safety drug profile [11]. There are multiple studies describing the radioprotective and radio-mitigative effects of Ciprofloxacin, through reduced bacterial infection and accelerated bone marrow recovery [12,13,14]. Captopril is an FDA-approved antihypertensive drug that has been used in patients to control blood pressure for the past 40 years [15]. However, this angiotensin-converting enzyme (ACE) inhibitor also possesses anti-inflammatory properties and alters the cell cycle of bone marrow stem cells [16,17]. Captopril protects from ARS when administered before or after whole-body irradiation by inhibiting the proliferation of bone marrow stem cells [18,19,20]. PrC-210 is an aminothiol, with a mechanism of action similar to Amifostine, but does not produce side effects, such as nausea or vomiting [21]. PrC-210 is a free radical scavenger and, when used prior to radiation, protects from DNA damage and improves survival against whole-body irradiation exposure [22,23]. 5-AED is a naturally occurring steroid molecule with estrogenic activity, and is known to elevate tissue levels of growth colony-stimulating factor (G-CSF) and interleukin-6 (IL-6) [24]. There is a wealth of data from multiple labs demonstrating that 5-AED significantly inhibits radiation damage [25,26,27]. The main mechanism of action for 5-AED in preventing radiation damage is the enhanced survival of hematopoietic progenitor cells [28]. The structurally related 5-AET also promotes survival after whole-body irradiation [25]. Both 5-AED and 5-AET have demonstrated safety in human trials [29].

Although there are data demonstrating the radioprotective properties of each of these selected compounds, the radiation sources and animal models used vary drastically from study to study and it is difficult to compare the efficacy of one drug to another. Thus, we set out to test these drugs using similar models so that a direct comparison can be made between the radioprotectors. In addition, we also developed a set of in vitro assays using GI and bone marrow progenitor cells to help screen the efficacy of radioprotectors and demonstrated that the efficacy measured in the in vitro assays correlated well with in vivo efficacy.

## 2. Results

### 2.1. Amifostine Protects Against Radiation Damage

For all the experimental data shown, the doses and routes of administration for each of the drugs tested were based on human or animal phamacokinetic data that have previously shown efficacy as a radioprotector. Therefore, we compare drugs at their known optimal dose, timing, and route of administration using the same in vitro and in vivo models to compare efficacy as radioprotectors.

**Figure 1:**
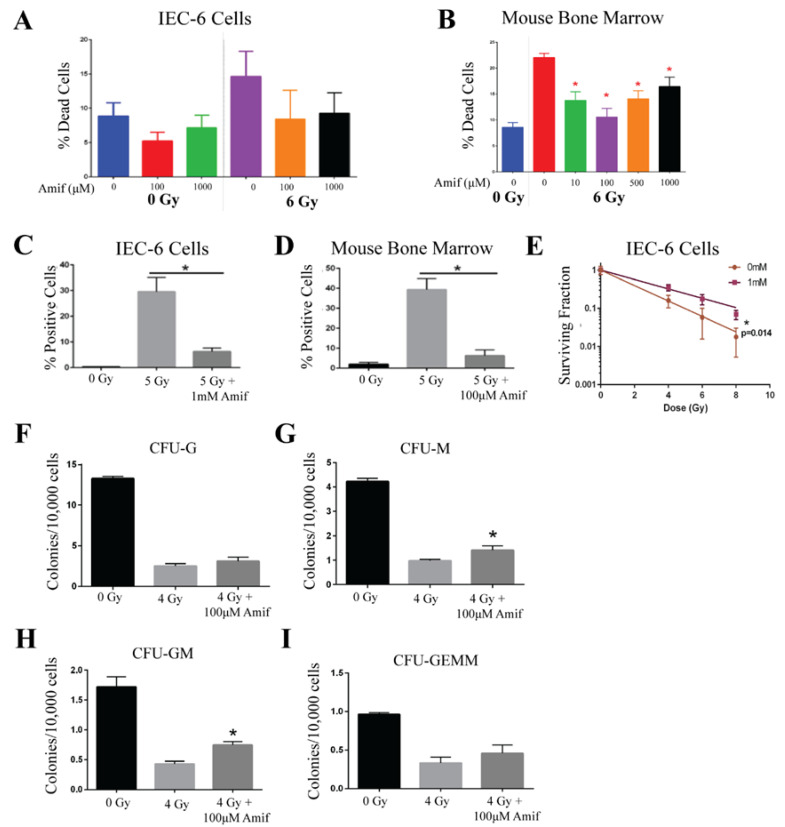
Amifostine protects rat intestinal cells and mouse bone marrow cells from acute radiation damage. (**A**) Amifostine (Amif) was administered to IEC-6 at doses of 100 and 1000 µM 30 min before exposure to 6 Gy of radiation, and cell death enumerated. (**B**) Cell death was quantified by viability in mouse bone marrow cells treated with various doses of Amif and 6 Gy of radiation. (**C**) IEC-6 cells and (**D**) mouse bone marrow cells were treated with 100 µM of Amif and 5 Gy of radiation to determine the accumulation of DNA damage via γ-H2AX staining. (**E**) A clonogenic assay of IEC-6 cells pre-treated with 1 mM of Amif 30 min before radiation was used to determine the ability of these cells to form colonies. (**F**) Colony formation units of granulocytes (CFU-G); (**G**) macrophages (CFU-M); (**H**) granulocyte, macrophage (CFU-GM); and (**I**) granulocyte, erythrocyte, macrophage, and megakaryocyte (CFU-GEMM) progenitors were assayed in mouse bone marrow cells treated with 100 µM of Amif 30 min before receiving 4 Gy of radiation. All experiments were executed three times. * Denotes a significant difference (*p* ≤ 0.05) as compared to the untreated, irradiated control.

Amifostine, an FDA-approved radioprotector used in cancer patients, was a positive control for the development of the in vitro assays to test the efficacy potential of ARS radioprotectors. Amifostine was administered to IEC-6 cells, a rat epithelial cell line, or primary mouse bone marrow cells for 30 min before being removed from the media, as this is the half-life of the drug in mice. Immediately after Amifostine treatment, cells were exposed to radiation. Water was used as the control (0 µM) treatment. To assess the toxicity of Amifostine and to determine the highest possible dose providing protection from radiation, a viability assay was conducted using a range of doses (10 µM, 100 µM, 500 µM, and 1000 µM) and 6 Gy of radiation. In the IEC-6 cells, no toxicity was observed in the cells treated with Amifostine prior to radiation; however, Amifostine was not able to significantly reduce radiation-induced cell death [Figure 1A]. Alternatively, all doses of amifostine administered to mouse bone marrow cells were able to protect the cells from radiation-induced cell death [Figure 1B]. The most effective dose of Amifostine was 1000 µM for the IEC-6 cells and 100 µM for the mouse bone marrow cells, which were used for all remaining assays.

Since radiation is known to damage DNA, γ-H2AX was quantified via immunofluorescence to determine the ability of Amifostine to reduce this known marker of DNA damage. IEC-6 cells were treated with 1000 µM and mouse bone marrow cells with 100 µM of Amifostine for 30 min before exposure to 5 Gy. DNA damage was significantly induced by radiation exposure and attenuated by Amifostine treatment in both the IEC-6 cells [Figure 1C] and the mouse bone marrow cells [Figure 1D]. Accumulation of DNA damage inhibits the survival and regeneration of stem cells in the GI and bone marrow. To this extent, a colony formation assay was conducted using 0 Gy, 4 Gy, 6 Gy, and 8 Gy of radiation. Amifostine protected IEC-6 cells from the loss of clonogenicity after radiation [Figure 1E]. Colony formation units (CFUs) were quantified in mouse bone marrow cells and classified as coming from one of four types of progenitor cells: CFU-G (granulocyte), CFU-M (macrophage), CFU-GM (granulocyte, macrophage), or CFU-GEMM (granulocyte, erythrocyte, macrophage, and megakaryocyte). Radiation was determined to significantly reduce the ability of the bone marrow cells to form colonies. Amifostine did not protect the colony formation of CFU-G or CFU-GEMM [Figure 1F,I]. However, significant protection was observed in the CFU-M and CFU-GM populations [Figure 1G,H]. Overall, Amifostine can attenuate radiation-induced toxicities, such as cell death, DNA damage, and reduced clonogenicity, which correlates well with the observed radioprotective capability of Amifostine in vivo [9,10]. Because Amifostine is a well-described radioprotector and FDA-approved [30], there was no need to further validate our findings in vivo.

### 2.2. Ciprofloxacin Does Not Protect Against Radiation-Induced Toxicities

**Figure 2:**
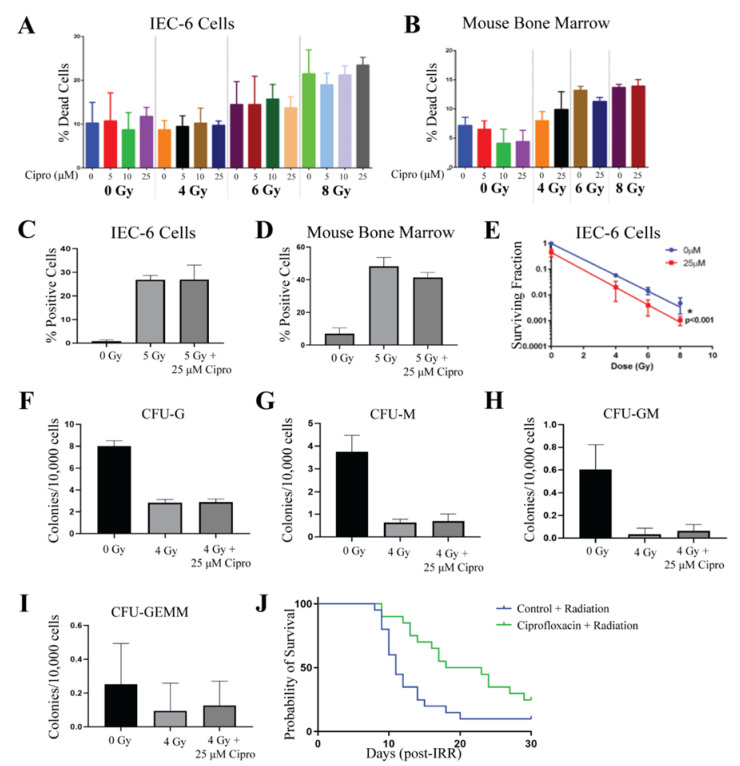
Ciprofloxacin does not protect rat intestinal cells and mouse bone marrow cells from acute radiation damage. (**A**) Quantification of cell death was observed in IEC-6 cells treated with 5, 10, or 25 µM of Ciprofloxacin (Cipro) one hour before exposure to 4, 6, or 8 Gy. (**B**) Cell death was quantified in mouse bone marrow cells treated with various concentrations of Cipro. (**C**) DNA damage was assessed by enumerating γ-H2AX^+^ IEC-6 cells or (**D**) mouse bone marrow cells treated with 25 µM of Cipro. (**E**) Clonogenic capacity was measured after administering 25 µM of Cipro to IEC-6 cells one hour before radiation. (**F**) Colony formation units of granulocytes (CFU-G); (**G**) macrophages (CFU-M); (**H**) granulocyte, macrophage (CFU-GM); and (**I**) granulocyte, erythrocyte, macrophage, and megakaryocyte (CFU-GEMM) progenitors were quantified in mouse bone marrow cells treated with Cipro (25 µM) and 4 Gy. Three replicates were completed from each assay. (**J**) Mice were administered 0.67 mg/mL of Cipro 1 h before whole-body irradiation (7.3 Gy) over 14 days after radiation. The overall 30-day survival was not significantly enhanced by Cipro treatment. Twenty mice per group were used to evaluate survival.

Ciprofloxacin, a fluoroquinolone antibiotic, has been identified as a potential radioprotector due to its antimicrobial function and ability to enhance bone marrow recovery rates [12]. The viability of IEC-6 and mouse bone marrow cells was evaluated using Ciprofloxacin at doses of 5 µM, 10 µM, or 25 µM. Drug or 0.1N HCl (control, 0 µM) was added to the cell culture media and incubated with the cells for 1 h before exposure to 0 Gy, 4 Gy, 6 Gy, or 8 Gy of radiation. No toxicity was observed in the IEC-6 cells or bone marrow cells when treated with the above doses of Ciprofloxacin at 0 Gy. Additionally, there was no observed protection from radiation-induced cell death in IEC-6 [Figure 2A] or bone marrow cells [Figure 2B].

IEC-6 cells were treated with 0 µM or 25 µM of Ciprofloxacin for 1 h and then irradiated with 5 Gy. DNA damage was assessed by quantifying the amount of cells that stained positive for γ-H2AX. Radiation induced significant DNA damage within the IEC-6 cells; however, Ciprofloxacin was not able to protect against this damage [Figure 2C]. Bone marrow cells were treated with 25 µM of Ciprofloxacin for 1 h before exposure to 5 Gy. Like in the IEC-6 cells, Ciprofloxacin was unable to attenuate DNA damage accumulation in the bone marrow cells [Figure 2D]. Colony formation was observed in IEC-6 cells treated with and without 25 µM of Ciprofloxacin and 4 Gy, 6 Gy, and 8 Gy. Ciprofloxacin did not protect IEC-6 cells from the loss of clonogenicity after radiation. Interestingly, 25 µM of Ciprofloxacin further suppressed the growth of colonies, causing a significant inhibition of colony formation when irradiated with 8 Gy [Figure 2E]. The assay was repeated with 5 µM of Ciprofloxacin, which resulted in no difference from the control cells. Furthermore, treating bone marrow cells with 25 µM of Ciprofloxacin did not significantly protect the cells from the radiation-induced loss of clonogenicity [Figure 2F,I].

To assess how Ciprofloxacin would behave as a radioprotector in vivo, CD2F1 mice were treated with Ciprofloxacin via drinking water (dw) at approximately 0.67 mg/mL/day; mice drank 4 mL/day for a dosage of 2.7 mg of Ciprofloxacin per day, or plain water as a control, starting one hour before radiation, 7.3 Gy, and continuing for 14 days following radiation. This dosing scheme mimics a course of antibiotics that would be administered to patients. Since manipulating animals by gavage after radiation causes stress that can result in animal death, Ciprofloxacin was administered through the drinking water rather than gavage. Although Ciprofloxacin enhanced the mean survival time, similar to the results of the in vitro assays, Ciprofloxacin did not significantly increase overall survival after radiation exposure [Figure 2J]. The results from this figure indicate that Ciprofloxacin does not behave as a radioprotector from ARS.

### 2.3. Captopril

**Figure 3:**
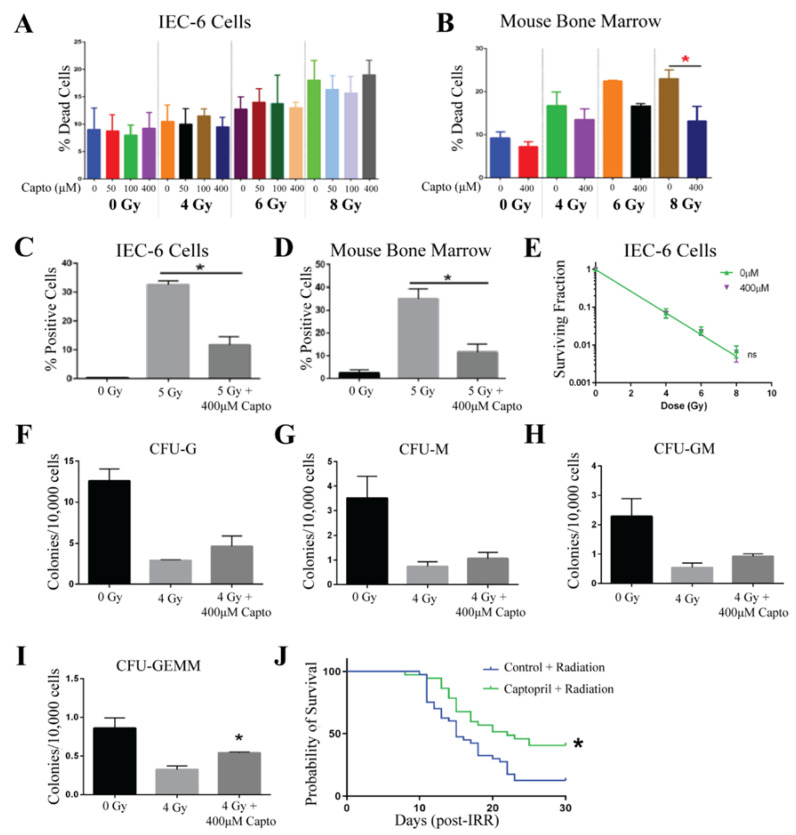
Captopril protects against ARS. (**A**). Toxicity and protection from radiation-induced cell death was evaluated in IEC-6 cells treated with 50, 100, or 400 µM one hour before exposure to 4, 6, or 8 Gy. (**B**) The percentage of dead cells was quantified in mouse bone marrow cells treated with Captopril (Capto, 400 µM). (**C**) DNA damage was quantified by enumerating γ-H2AX^+^ IEC-6 cells and (**D**) mouse bone marrow cells pre-treated with 400 µM of Capto and exposed to 5 Gy. (**E**) A clonogenic survival assay was completed on IEC-6 cells dosed with Capto (400 µM), or the control (0 µM), and radiation. To assay the colony formation of bone marrow cells, 400 µM of Capto was administered to the cells 1 h before radiation (4 Gy). (**F**) Colony formation units of granulocytes (CFU-G); (**G**) macrophages (CFU-M); (**H**) granulocyte, macrophage (CFU-GM); and (**I**) granulocyte, erythrocyte, macrophage, and megakaryocyte (CFU-GEMM) progenitors were quantified 10 days after drug and radiation treatment. Three replicates were completed from each assay. * Indicates a significant difference (*p* ≤ 0.05) as compared to the untreated, irradiated control. (**J**) Mice were administered 110 mg/mL of Capto 48, 24, and 1 h before whole-body irradiation (7.3 Gy). The overall 30-day survival was significantly enhanced by Capto treatment. Forty mice per group were used to evaluate survival.

Captopril was evaluated at doses of 50 µM, 100 µM, and 400 µM, where it was added to the cell culture medium one hour prior to radiation exposure. In IEC-6 cells, viability was not affected by Captopril at this range of doses. However, there was also no protection from radiation-induced cell death [Figure 3A]. For the remaining assays, 400 µM of Captopril was used. At this concentration, Captopril was able to protect mouse bone marrow cells from radiation-induced cell death when administered to the cells 1 h before radiation [Figure 3B]. In both IEC-6 cells and bone marrow cells, treatment with 400 µM of Captopril reduced the amount of γ-H2AX staining observed when cells were irradiated with 5 Gy [Figure 3C,D]. Colony formation was assessed in the IEC-6 cells using a clonogenic assay. As above, the cells were treated with 400 µM of Captopril 1 h before radiation exposure. Cells were then treated with 0, 4, 6, or 8 Gy. Similar to the viability assay, no difference was determined in the clonogenicity of the IEC-6 cells when treated with or without Captopril [Figure 3E]. When Captopril (400 µM) was administered to mouse bone marrow cells 1 h before exposure to 4 Gy of radiation, the colony formation of most of the bone marrow progenitor cells was not protected against radiation exposure [Figure 3F–H]. Interestingly, a significant difference was observed in the GEMM population, the most stem-like progenitor cell population [Figure 3I].

In humans, Captopril is administered as a pill two to three times a day, due to its short half-life. To mimic oral administration, we first tried to administer Captopril via drinking water; however, the CD2F1 mice would not drink Captopril-treated water. C57Bl/6 mice drank Captopril-treated water, so we switched strains for this compound. However, the drinking amounts were not consistent between groups after radiation. Therefore, we switched drug administration routes and instead gavaged mice three times over a 48 h period before exposing the mice to radiation. Gavage is a more accurate way to administer a known concentration, and because Captopril was administered only before radiation, we did not handle mice after radiation, which could have induced more stress contributing to animal death. To test the ability of Captopril to protect against ARS in vivo, mice were administered Captopril at a dose of 110 mg/kg three times via oral gavage (gav) at 48, 24, and 1 h before receiving 7.3 Gy of total body irradiation. In the control mice, an equal volume of water was gavaged 48, 24, and 1 h prior to total body irradiation. After 30 days, the overall survival of the Captopril-treated mice was 40.5% (15/37 mice), a significant increase as compared to the overall survival of 12.5% (5/40 mice) in the control-treated mice [Figure 3J]. From these results, we conclude that Captopril protects against radiation-induced toxicities and when administered in three doses of 110 mg/kg can reduce 30-day mortality and extend the mean survival time. We also repeated this dosing regimen in CD2F1 mice and found a significant 27% increase in animal survival, which is similar to the 28% survival observed for C57Bl/6 mice.

### 2.4. PrC-210

**Figure 4:**
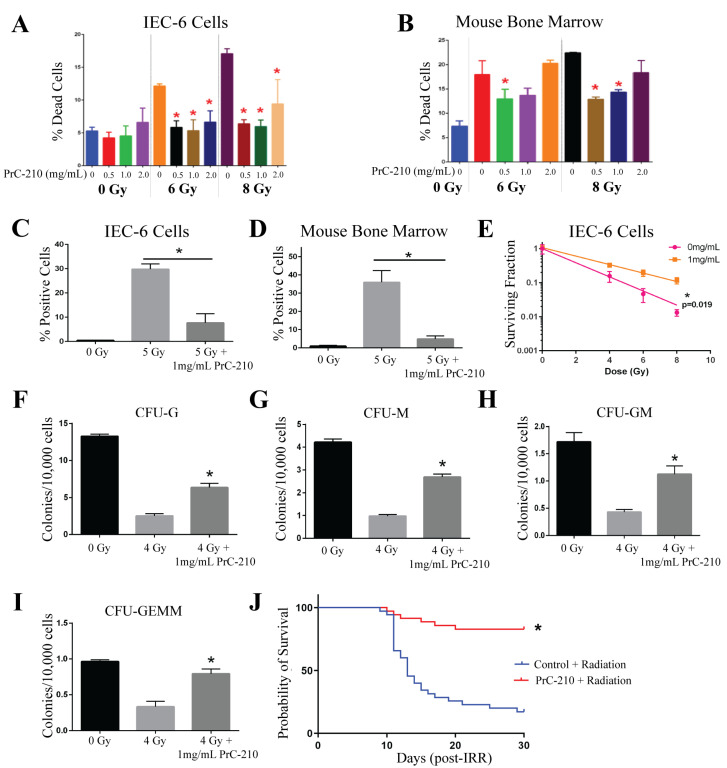
PrC-210 protects against ARS both in vitro and in vivo. (**A**) The viability of IEC-6 cells and (**B**) mouse bone marrow cells treated with 0.5, 1, or 2 mg/mL thirty minutes before exposure to 6 or 8 Gy was assessed as the percentage of dead cells. (**C**) DNA damage, indicated by γ-H2AX staining, in IEC-6 cells and (**D**) mouse bone marrow cells, pre-treated with 1 mg/mL of PrC-210 before exposure to 5 Gy. (**E**) A total of 1 mg/mL of PrC-210, or control, was administered to IEC-6 cells prior to 4, 6, and 8 Gy of radiation exposure when compared to assessments of the control and colony formation. To assay the colony formation of bone marrow cells, 1 mg/mL of PrC-210 was administered to the cells 30 min before radiation (4 Gy). (**F**) Colony formation units of granulocytes (CFU-G); (**G**) macrophages (CFU-M); (**H**) granulocyte, macrophage (CFU-GM); and (**I**) granulocyte, erythrocyte, macrophage, and megakaryocyte (CFU-GEMM) progenitors were quantified in mouse bone marrow cells treated with PrC-210 (1 mg/mL) prior to radiation exposure (4 Gy). Three biological replicates were completed from each assay. (**J**) Mice were administered 900 mg/kg of PrC-210 30 min before whole-body irradiation (7.3 Gy). The overall 30-day survival was significantly enhanced by PrC-210 treatment. Thirty-five mice per group were used to evaluate survival. * Indicates a significant difference (*p* ≤ 0.05) compared to the untreated, irradiated control.

PrC-210, a free radical scavenger, was tested at concentrations of 0.5, 1, and 2 mg/mL. IEC-6 or mouse bone marrow cells were treated with PrC-210, or water (control), 30 min before radiation exposure as this is the half-life in mice. The viability of IEC-6 cells indicates that PrC-210 is not toxic to intestinal cells at the administered doses. Impressively, PrC-210 protected against radiation-induced cell death at both 6 Gy and 8 Gy [Figure 4A]. In the mouse bone marrow cells treated with PrC-210, mild toxicity was observed with the 2 mg/mL dose; however, there was significant protection from radiation-induced cell death at 0.5 and 1 mg/mL [Figure 4B]. For the remaining in vitro assays, 1 mg/mL of PrC-210 was used. To test the ability of PrC-210 to reduce DNA damage, cells positive for γ-H2AX were quantified. In IEC-6 cells, γ-H2AX staining was highly elevated when the cells were exposed to 5 Gy of radiation, and when treated with PrC-210 30 min before radiation, IEC-6 cells had significantly fewer γ-H2AX^+^ cells [Figure 4C]. In the mouse bone marrow cells, PrC-210 treatment also attenuated DNA damage induced at 5 Gy [Figure 4D]. In IEC-6 cells, 1 mg/mL of PrC-210 given 30 min before radiation significantly protected cells and allowed for enhanced colony formation [Figure 4E]. Colonogenicity of mouse bone marrow cells was protected, as evidenced by the increased formation of all types of mouse bone marrow progenitor cells: granulocytes (CFU-G) [Figure 4F]; macrophages (CFU-M) [Figure 4G]; granulocyte, macrophage (CFU-GM) [Figure 4H]; and granulocyte, erythrocyte, macrophage, megakaryocyte (CFU-GEMM) [Figure 4I]. PrC-210 worked as a radioprotector in all six in vitro assays.

To evaluate the ability of PrC-210 to behave as a radioprotector in vivo, male CD2F1 mice were administered 900 mg/kg of PrC-210 intraperitoneally (ip) 30 min prior to 7.3 Gy of total body irradiation. An equivalent volume of water was injected 30 min before radiation in control mice. In this model, PrC-210 significantly increased the overall number of surviving mice after 30 days from 17% (6/35 mice) in control mice to 83% (29/35 mice) in PrC-210-treated mice [Figure 4J]. Notably, PrC-210 was able to protect against radiation-induced cell death, increase DNA damage, decrease clonogenicity in both IEC-6 cells and the bone marrow, and significantly reduce 30-day mortality in vivo.

### 2.5. 5-AED

**Figure 5:**
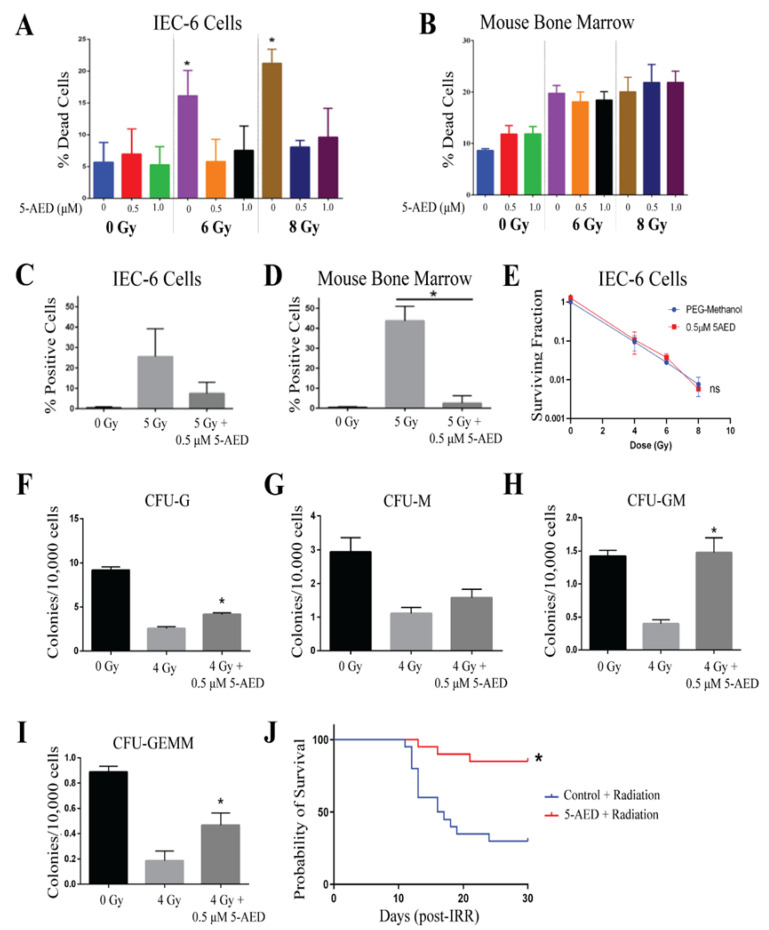
5-AED is a radioprotector in vitro and in vivo. (**A**) Radiation-induced cell death in IEC-6 cells treated with 0, 0.5, or 1 µM of 5-AED one hour before exposure to 6 or 8 Gy. (**B**) Quantification of cell death in mouse bone marrow cells treated with 5-AED. (**C**) DNA damage in IEC-6 cells treated with or without 5-AED (0.5 µM) and 5 Gy of radiation was assessed by enumerating the percentage of cells positive for γ-H2AX staining. (**D**) Percentage of γ-H2AX^+^ mouse bone marrow cells treated with or without 0.5 µM of 5-AED and 5 Gy. (**E**) Clonogenic capacity of IEC-6 cells was evaluated at 4, 6, and 8 Gy when treated with control (PEG-Methanol) or 0.5 µM of 5-AED. (**F**) Colony formation units of granulocytes (CFU-G); (**G**) macrophages (CFU-M); (**H**) granulocyte, macrophage (CFU-GM); and (**I**) granulocyte, erythrocyte, macrophage, and megakaryocyte (CFU-GEMM) progenitors were quantified 10 days after bone marrow cells were treated with 5-AED (0.5 µM) one hour prior to radiation exposure (4 Gy). Three biological replicates were completed from each assay. * Indicates a significant difference (*p* ≤ 0.05) from the untreated, irradiated control. (**J**) Mice were administered 30 mg/kg of 5-AED 24 h before whole-body irradiation (7.3 Gy). 5-AED treatment significantly protected the overall 30-day survival. Twenty mice per group were used to evaluate survival.

5-AED was evaluated at doses of 0.5 and 1 µM, 1 h before radiation treatment. 5-AED was reconstituted in equal parts of methanol and PEG-400, which was utilized as a control. Cells, IEC-6, and mouse bone marrow, were treated with 5-AED or control 1 h before radiation. 5-AED did not show any toxicity at these doses. Radiation-induced cell death was diminished when IEC-6 cells were pre-treated with 5-AED [Figure 5A]. The same attenuation of radiation-induced cell death was not observed in mouse bone marrow cells treated with 5-AED [Figure 5B]. Given the results of the IEC-6 viability, 0.5 µM of 5-AED was used for the rest of the assays. In IEC-6 cells, 5-AED did reduce DNA damage accumulation, as indicated by γ-H2AX^+^ cells, following 5 Gy of radiation; however, the effect was not significant [Figure 5C]. Conversely, 5-AED did significantly reduce the DNA damage induced by 5 Gy of radiation in mouse bone marrow cells [Figure 5D].

5-AED did not protect the IEC-6 cells from the reduction in clonogenicity that was observed with 4, 6, and 8 Gy of radiation [Figure 5E]. In mouse bone marrow cells, the formation of the granulocyte (CFU-G) was reduced with 4 Gy of radiation, but significantly increased when the cells were treated with 0.5 µM of 5-AED 1 h before radiation [Figure 5F]. The formation of the macrophage (CFU-M) progenitor population was not significantly altered by 5-AED treatment [Figure 5G]. Granulocyte, macrophage (CFU-GM), and granulocyte, erythrocyte, macrophage, megakaryocyte (CFU-GEMM) populations were protected from 4 Gy of radiation by 0.5 µM of 5-AED. Overall, 5-AED protected mouse bone marrow colony formation against radiation, but did not protect IEC-6 cells.

Male CD2F1 mice were administered 30 mg/kg of 5-AED subcutaneously (sc) 24 h prior to radiation exposure, as this was previously reported to be efficacious [26]. An equivalent volume (100 µL) of methanol:PEG-400 (50:50) was injected into control mice 24 h before radiation. After 30 days, mice treated with 5-AED showed significantly higher overall survival (85%, 17/20 mice) when compared to control animals treated with radiation and vehicle (30%, 6/20 mice). Mean survival time (MST) could not be compared as more than 50% of the mice survived past 30 days. In the control mice, the MST was 16.5 days. Overall, 5-AED significantly protected from ARS-induced lethality.

### 2.6. 5-AET

**Figure 6:**
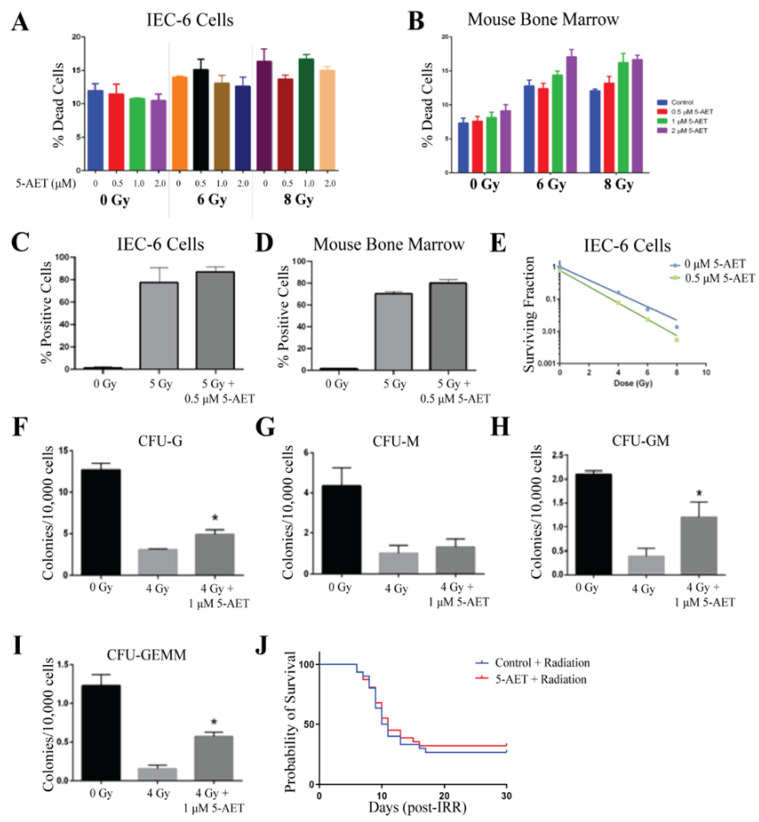
5-AET does not protect against radiation damage. (**A**) Radiation-induced cell death in IEC-6 cells treated with 0, 0.5, 1, or 2 µM of 5-AET one hour before exposure to 6 or 8 Gy. (**B**) Quantification of cell death in mouse bone marrow cells treated with 5-AET. (**C**) DNA damage in IEC-6 cells treated with or without 5-AET (0.5 µM) and 5 Gy of radiation was assessed by enumerating the percentage of cells positive for γ-H2AX staining. (**D**) Percentage of γ-H2AX^+^ mouse bone marrow cells treated with or without 0.5 µM of 5-AET and 5 Gy. (**E**) Clonogenic capacity of IEC-6 cells was evaluated at 4, 6, and 8 Gy when treated with control (PEG-Methanol) or 0.5 µM of 5-AET. (**F**) Colony formation units of granulocytes (CFU-G); (**G**) macrophages (CFU-M); (**H**) granulocyte, macrophage (CFU-GM); and (**I**) granulocyte, erythrocyte, macrophage, and megakaryocyte (CFU-GEMM) progenitors were quantified 10 days after bone marrow cells were treated with 5-AET (1 µM) one hour prior to radiation exposure (4 Gy). Three biological replicates were completed from each assay. * Indicates a significant difference (*p* ≤ 0.05) from the untreated, irradiated control. (**J**) Mice were administered 30 mg/kg of 5-AET 24 h before whole-body irradiation (7.3 Gy). 5-AET treatment did not significantly alter the overall 30-day survival. Twenty mice per group were used to evaluate survival.

The detrimental effects of radiation were evaluated when cells were treated with and without 5-AET at doses of 0.5, 1, and 2 µM 1 h before radiation. Similar to 5-AED, 5-AET was prepared as a solution in equal parts methanol and PEG-400. Therefore, methanol:PEG-400 was used as a control (0 µM). Viability was not enhanced in either IEC-6 or mouse bone marrow cells when treated with any dose of 5-AET and either 6 Gy or 8 Gy of radiation [Figure 6A,B]. Interestingly, for bone marrow cells, cell death was slightly enhanced from radiation with the highest dose of 5-AET, so 0.5 µM and 1 µM of 5-AET were utilized for the remaining assays. IEC-6 cells and bone marrow cells had elevated levels of DNA damage, as indicated by γ-H2AX staining, when treated with 5 Gy of radiation. Treatment of the cells with 0.5 µM of 5-AET 1 h before radiation did not decrease this DNA damage [Figure 6C,D]. Thus, the lack of protection from radiation-induced cell death can be attributed to an increase in DNA damage.

Even though 5-AET did not protect against radiation-induced cell death or the increase in DNA damage caused by radiation, we still evaluated 5-AET for its ability to protect the clonogenicity of IEC-6 cells and bone marrow cells. 5-AET did not protect IEC-6 cells from the loss of clonogenicity after radiation with 4, 6, or 8 Gy [Figure 6E]. Protection of colony formation was evident in almost all types of mouse bone marrow progenitor cells, including CFU-G, CFU-GM, and CFU-GEMM; CFU-M formation was not significantly enhanced with 5-AET [Figure 6F,I]. The colony formation of bone marrow cells was the only assay where radioprotection was observed. In all other assays, 5-AET did not show any significant difference from the control.

Twenty-four hours prior to total body irradiation (7.3 Gy), mice were subcutaneously dosed with 30 mg/kg of 5-AET, as previously reported [25]. 5-AET was dissolved in equal amounts of methanol and PEG-400; therefore, methanol:PEG-400 served as the vehicle control. The overall 30-day survival was 35% in the 5-AET-treated mice and 30% in control mice [Figure 6J]. Therefore, compared to the vehicle control, 5-AET did not behave as a radioprotector when used in vitro or in vivo.

### 2.7. In Vitro Assays Predict In Vivo Survival

**Figure 7:**
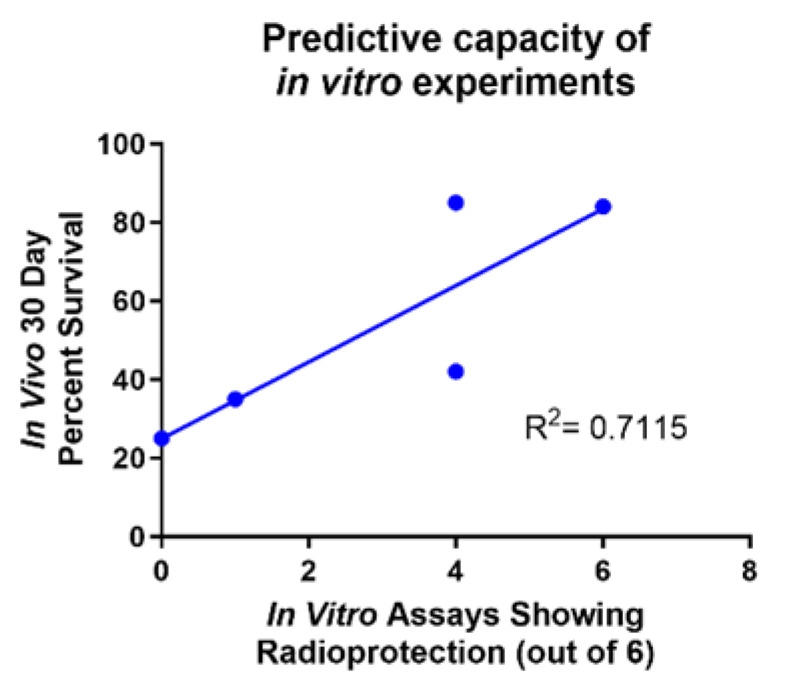
Predictive capacity of in vitro experiments. Comparison of the number of in vitro assays showing radioprotection versus the in vivo 30-day percent survival. Captopril and 5-AED are the two outlier compounds. R^2^ = 0.7115.

One of the goals of this study was to establish a method of screening for the efficacy of potential ARS protectors before administering the compounds to mice. Throughout this study, we modeled common ARS symptoms in both GI progenitor epithelial cells (IEC-6) and primary mouse bone marrow cells to test radioprotection behavior in vitro and compared those results to overall in vivo protection [Table 1]. Viability, DNA damage (γ-H2AX) and colony formation were the experimental endpoints assayed in vitro using Amifostine as a positive control. A noticeable trend was observed: the compound exhibited a significant increase in overall survival in vivo following radiation exposure if the compound was also radioprotective in all six in vitro assays [Figure 7]. Captopril and 5-AED were the only two compounds that did not fall directly on the trend line. Both compounds showed radioprotection in four of the six in vitro assays; however, 5-AED behaved much better in vivo than Captopril. While these six in vitro assays do not perfectly convey in vivo behavior, they provide a way to identify compounds that are highly likely to be radioprotectors. Overall, these assays can be used as a method to screen potential radioprotectors for their ability to protect against ARS.

## 3. Discussion

Whole-body high-dose radiation is responsible for the induction of ARS. To combat radiation-induced cellular damage, radioprotectors are needed to mitigate the damage to regenerating stem cell populations. In this manuscript, we compared five previously demonstrated radioprotectors, Ciprofloxacin, Captopril, PrC-210, 5-AED, and 5-AET to the only FDA-approved radioprotector, Amifostine, for their ability to protect rat epithelial intestinal cells (IEC-6 cells), primary mouse bone marrow cells, and whole-body irradiated mice from radiation damage. To our knowledge, this is the first study to conduct a head-to-head comparison of these known radioprotectors.

Overall, of the five compounds, we identified PrC-210 as the best candidate for a radioprotector. In the in vitro assays, PrC-210 was able to significantly reduce radiation-induced cell death, DNA damage, and increase the clonogenicity of the cells treated with radiation. In vivo, PrC-210 increased the overall 30-day survival from 15% to 85%. 5-AED was the next-best radioprotector, having behaved effectively in reducing cell death in IEC-6 cells and bone marrow cells, reducing DNA damage, and protecting colony formation. However, in the IEC-6 cells, DNA damage and colony formation were not protected, and in the bone marrow cells, radiation-induced cell death was not protected. Nonetheless, significant protection was observed in mice treated with 5-AED twenty-four hours before radiation exposure. Like 5-AED, Captopril also protected the IEC-6 cells and bone marrow cells in some but not all of the in vitro assays, but reduced 30-day mortality and extended the mean survival time of mice exposed to 7.3 Gy of whole-body irradiation in vivo. 5-AET only showed protection in the clonogenicity of mouse bone marrow cells and, as expected, did not significantly increase the 30-day survival of the irradiated mice. There are only two papers in the literature testing 5-AET against ARS. One paper by Loria et al. demonstrated that, in combination with infection, 5-AET was a protector against ARS [25]. This paper demonstrated that 5-AET protected mice from ARS and coxsackievirus, by increasing CD4^+^ and CD8^+^ cells in the spleen. However, another study by Whitnall et al. showed that 5-AET alone did not protect mice from ARS when administered prophylactically without an infection [26]. Our data support Whitnall et al.’s findings. Lastly, Ciprofloxacin did not show any protection in the in vitro assays, and although Ciprofloxacin increased the mean survival time, by day 30, there was no significant increase in overall survival in vivo.

Currently, there are no in vitro assays that fully predict how radioprotectors will behave once inside a living organism. To address this, we investigated a series of in vitro assays to determine the overall radiation-induced cell death, DNA damage, and colony formation in IEC-6 cells and mouse bone marrow cells. Based on the data presented in this manuscript, we can predict the likely success of these radioprotectors in vivo. While these six in vitro assays do not confer a perfect prediction of in vivo survival, they do offer a solid indication [Figure 7].

The limitations of the in vitro experiments of this study are that if in vivo drug metabolism is required to generate the active molecule, the drug may not be efficacious in vitro. Additionally, if a drug works on other cell types in the body besides bone marrow or gastrointestinal cells, the drug would appear not to be efficacious using the in vitro assays but may work well in vivo. Our data indicate that if protection is observed in vitro on hematopoietic and GI stem cells, it is likely to be protective in vivo. We did not investigate the combination of these drugs to be administered as a cocktail before radiation exposure. This is a future direction as these compounds have different mechanisms of action and may work even better when used as a combinational therapy. IEC-6 cells behave differently after they have been cultured, which results in more variability in the experimental endpoints. We did not use cells after passage 12 to limit the experimental variability. Only male mice were used in our study, and there could have been sex bias with some of the compounds used; therefore, female mice should also be used in future studies to discern any differences in efficacy between the sexes. Lastly, ARS can take the form of both gastrointestinal ARS (GI ARS) and hematopoietic ARS (hARS). In our in vivo model, we only investigated hARS endpoints, which could explain why some of the compounds performed better in one cell type compared to the other. Further studies evaluating GI health need to be conducted to determine if these compounds have any GI ARS-mitigating potential. Additionally, our study concluded 30 days post-injection, neglecting any delayed effects of ARS, such as lung, kidney, or heart failure [31]. 

## 4. Materials and Methods

### 4.1. Cell Culture

IEC-6 rat GI progenitor epithelial cells were obtained from ATCC (CRL-1592, Manassas, VA, USA). IEC-6 cells were maintained in a DMEM medium containing 10% fetal bovine serum (FBS), 1% penicillin/streptomycin, and 1 mg of insulin. Cultures were kept in an incubator at 37 °C with 5% CO_2_. The IEC-6 cell line was kept through passage 12.

### 4.2. Experimental Animals

C57Bl/6 male mice (aged 12–14 weeks) were obtained from Jackson Laboratories (Bar Harbor, ME, USA). CD2F1 male mice (aged 12–14 weeks) were obtained from Charles River (Wilmington, MA, USA). All mice were housed at the University of Nebraska Medical Center (UNMC) in accordance with the Guide for Care and Use of Laboratory Animals by the National Institutes of Health. Mice were kept on a 12 h light/dark cycle and fed and watered ad libitum. Animal treatment procedures were approved and enforced by the UNMC Institutional Animal Care and Use Committee (22-034-07-FC).

### 4.3. Molecules Tested

Amifostine, Captopril, and Ciprofloxacin were obtained from Sigma-Aldrich (St. Louis, MO, USA). PrC-210 was obtained from the Wisconsin Army Research Foundation. 5-AED and 5-AET were prepared through the reported methods [32].

### 4.4. Mouse Bone Marrow Isolation

Male CD2F1 mice were euthanized according to the IACUC protocol. Femurs and tibias were collected from each mouse. Bones were cleaned by removing the skin and muscular tissues. The ends of the femur and tibia were cut and a 25G needle with a syringe filled with HBSS (+5% FBS) was used to flush out the bone marrow. Flushing was repeated 3 times. The isolated bone marrow was passed through a cell strainer to remove any clumps of dead cells. Live, white blood cells were counted using a hemocytometer with trypan blue.

### 4.5. Viability for IEC-6 Cells

GI Cells: IEC-6 cells were seeded in a six-well plate. The seeding density was 5 × 10^5^ cells/well. The next day, cells were treated with the test article or with respective controls and then irradiated with 0 Gy, 4 Gy, 6 Gy, and 8 Gy. Five days after radiation, cells were trypsinized, centrifuged, and re-suspended in 2 mL of fresh media. A total of 10 µL of cell suspension was mixed with 10 µL of trypan blue dye and loaded onto a hemocytometer to count live and dead cells. Additionally, viability was determined by loading 200 µL of cell suspension on a Vi-CELL BLU automated cell viability analyzer, per the manufacturer’s instructions.

Mouse Bone Marrow: Immediately after isolation, mouse bone marrow cells were treated with the test article or with respective controls and then irradiated with 0 Gy, 4 Gy, 6 Gy, and 8 Gy. After irradiation, 8 × 10^4^ cells/well were seeded in a 96-well plate in 200 µL of complete media (RPMI + 10% FBS + 10 ng/mL IL-6 + 10 ng/mL IL-3 + 20 ng/mL SCF). A total of 48 h after radiation, 10 µL of cell suspension was mixed with 10 µL of trypan blue dye and loaded on the hemocytometer to count live (white) and dead (blue) cells.

For both cell types, the % cell death was calculated as:

(1) $$\%\;\mathrm{cell}\;\mathrm{death}=\frac{\;\#\;\mathrm{dead}\;\mathrm{cells}}{\#\;\mathrm{total}\;\mathrm{cells}}\times 100$$

### 4.6. DNA Damage (γ-H2AX Staining)

GI Cells: IEC-6 cells were seeded into T-25 flasks with 2 × 10^5^ cells/flask, and the following day were treated with the test article followed by 5 Gy radiation. The flasks were returned to the incubator for 30 min, then detached with trypsin and counted. For each condition, 1.2 × 10^5^ cells were centrifuged and resuspended in 200 µL of FBS and then processed for immunofluorescence staining.

Mouse Bone Marrow: Mouse bone marrow cells were harvested, counted, and divided into microcentrifuge tubes with 1.2 × 10^5^ cells in 200 µL of media, then immediately treated with the test article and irradiated with 5 Gy radiation. After irradiation, the tubes were returned to the incubator for 30 min and then processed for immunofluorescence staining.

Immunofluorescence Procedure and Analysis: A Cytospin was used to apply 100 µL (or 6 × 10^4^ cells) to a negatively charged slide by centrifugation at 800× *g* for 3 min. The slides were allowed to dry for at least 30 min at room temperature, then fixed in 4% paraformaldehyde for 12 min. After fixation, the slides were washed for 10 min in PBS under stirring. Next, the cells were permeabilized with 0.5% *v/v* Triton X-100 in PBS for 8 min, with stirring, followed immediately by 15 min of washing in PBS. The slides were transferred to a humidified chamber, and 100 µL of blocking buffer (10% goat serum in 1× PBS) was applied to the cells for one hour at room temperature. Excess blocking buffer was blotted from the slide and replaced with 70 µL of the appropriate primary antibody (For IEC-6: Abcam (Cambridge, UK); cat. ab26350; 1:200, for mouse bone marrow: Abcam; cat. ab11174; 1:250) diluted in blocking buffer. The slides were incubated in a humidified chamber at 37 °C for 1 h, then washed thrice for 5 min each in PBS. Secondary antibodies diluted 1:500 in the blocking buffer (for IEC-6: GtαMs AlexaFluor488; cat. A11001; for mouse bone marrow: GtαRb AlexaFluor488; cat. A11008) were applied to the cells and the slides were incubated in a humidified chamber in the dark for 1 h at room temperature. The slides were washed three times for 5 min in PBS, then a coverslip was applied with DAPI mounting medium. The slides were imaged using a Leica DM4000B LED microscope; approximately 5 fields with the 20× objective were captured per slide. Images were exported to ImageJ version 1.48 for analysis. Total cells (DAPI/nuclei) and cells positively stained for γH2AX were counted in the field of view (5 fields/sample).

### 4.7. Clonogenic Survival

One day prior to treatment, IEC-6 cells were seeded into T-25 flasks at 3 × 10^5^ cells/flask. The following day, half of the cell cultures were treated with the test article, and the other half were treated with a control solvent. Pairs of flasks were then irradiated with either 0 Gy (sham), 4 Gy, 6 Gy, or 8 Gy. Immediately after radiation, the cells were detached and counted, then serially diluted to an appropriate concentration. Cells were seeded in triplicate for each group on 6-well plates according to the following plan:
**Treatment****Number of Cells Seeded**0 Gy 5004 Gy10006 Gy20008 Gy4000

Colonies were allowed to grow for 7–9 days until >50 cells were observed per colony. The colonies were fixed to the plate with 70% ethyl alcohol and then stained with 0.5% Crystal Violet in 25% methanol. Colonies were enumerated using a dissecting microscope, and the control plating efficiency was calculated using the 0 Gy + control solvent condition. The surviving fraction of each group was then normalized to the control plating efficiency and plotted on a semi-log graph.

(2) $$\mathrm{Plating}\;\mathrm{Efficiency}\;\left(\mathrm{PE}\right)=\frac{\#\;\mathrm{colonies}\;\mathrm{formed}}{\#\;\mathrm{cells}\;\mathrm{seeded}}\times \;\mathrm{Surviving}\;\mathrm{Fraction}=\frac{\mathrm{PE}\;\mathrm{of}\;\mathrm{treatment}\;\mathrm{group}}{\mathrm{PE}\;\mathrm{of}\;\mathrm{control}\;\mathrm{group}}$$

### 4.8. Colony Formation

Mouse bone marrow cells were harvested, counted, and divided into microcentrifuge tubes with 3 × 10^5^ cells (for control) or 6 × 10^5^ cells (for irradiated) in 400 µL of media, then immediately treated with the test article or appropriate control solvent and irradiated with 4 Gy radiation. Immediately following irradiation, the cell suspension was added to 4 mL of room-temperature Methocult and vortexed for 30 s. Samples were allowed to set for 5 min to allow bubbles to rise to the top of the volume. Next, a blunt-ended needle was used to dispense 1 mL of the mixture into each of three 35 mm dishes for technical replicates. The dishes were allowed to incubate for 12 days and then colony-forming units (CFUs) were classified into one of four types of progenitor cell: CFU-G (granulocyte), CFU-M (macrophage), CFU-GM (granulocyte, macrophage), or CFU-GEMM (granulocyte, erythrocyte, macrophage, megakaryocyte). As different numbers of cells were used to inoculate the dishes, the final CFU counts were normalized per 10,000 cells originally used.

### 4.9. In Vivo Drug Treatment

Ciprofloxacin: Male CD2F1 mice, aged 14 weeks, were administered 0.67 mg/mL of Ciprofloxacin orally via drinking water. Mice were exposed to the treated water 1 h prior to 7.3 Gy of whole-body irradiation and maintained administration through 14 days after radiation exposure. Control mice received an equivalent volume (~4 mL) of untreated water dosed as that of the Ciprofloxacin-treated mice.

Captopril: Male C57Bl/6 mice, aged 12 weeks, or male CD2F1 mice aged 14 weeks, were treated with 110 mg/kg of Captopril via oral gavage 48 h, 24 h, and 1 h before receiving 7.3 Gy of total body irradiation. Control mice received an equivalent volume (100 µL) of water via oral gavage at 48, 24, and 1 h pre-radiation.

PrC-210: Male CD2F1 mice, aged 14 weeks, were administered 900 mg/kg of PrC-210 via intraperitoneal injection 30 min before whole-body irradiation exposure, 7.3 Gy. Control mice were injected with an equivalent volume (100 µL) of water 30 min before radiation.

5-AED: Male CD2F1 mice, aged 14 weeks, were subcutaneously injected with 30 mg/kg of 5-AED 24 h before exposure to 7.3 Gy of whole-body irradiation. Methanol:PEG-400 was administered as the control 24 h prior to radiation.

5-AET: Male CD2F1 mice, aged 14 weeks, were subcutaneously injected with 3-mg/kg of 5-AET 24 h prior to receiving 7.3 Gy of whole-body irradiation. Control mice received a subcutaneous injection of 100 µL of methanol:PEG-400 (1:1) 24 h before radiation.

### 4.10. Animal Radiation Treatments

Following the dosing as stated above, mice were humanely restrained within a 50 mL conical tube with air holes to keep the radiation plane consistent. Mice received 7.3 Gy of whole-body irradiation using a RadSource 2000 X-Ray Box Irradiator (Buford, GA, USA) with a dose rate of 1.2 Gy/minute.

### 4.11. Mean Survival Time

Following treatment with 7.3 Gy of whole-body irradiation, the mice were housed under routine husbandry to determine the proportion of mice that survived for 30 days post-radiation. Mice were monitored for survival once/day until signs that the animals may require early euthanasia appeared, then twice/day until day 30. Mean survival time among decedents was presented by group using Kaplan–Meier Survival Curves.

### 4.12. Statistics

For the clonogenic survival assay with IEC-6 cells, statistical analysis was performed by non-linear regression with least squares fit to generate an appropriate model curve for each data set. An F-test was used to determine if the curves were significantly different or if one curve could adequately fit both data sets. For viability, DNA damage, and colony formation assays, one-way ANOVA, followed by Tukey’s test for multiple comparisons was used to determine significance between treatment groups. All the experiments were repeated at least three times, and for mouse bone marrow data, a different animal was used for each experiment to obtain true biological replicates. Data are presented as mean ± standard deviation. To determine the mean survival time, logistic regression was used to compare overall 30-day survival between each treatment group and the control. Thirty-day mortality and log-rank (Cox regression) analyses for treatment comparisons were conducted using a two-tailed 5% significance level.

## Figures and Tables

**Table 1:** Overview of in vitro and in vivo results.

Drug Name	Experimental Endpoint	Concentration	Cell Types Used	Radiation Protection?
Amifostine	Viability	100, 1000 µM	IEC-6	NO
		10, 100, 500, 1000 µM	Mouse Bone Marrow	YES
	γ-H2AX	1 mM	IEC-6	YES
		100 µM	Mouse bone marrow	YES
	Clonogenicity/Colony Formation	1 mM	IEC-6	YES
		100 µM	Mouse bone marrow	YES
Ciprofloxacin	Viability	5, 10, 25 µM	IEC-6	NO
		25 µM	Mouse Bone Marrow	NO
	γ-H2AX	25 µM	IEC-6	NO
		25 µM	Mouse bone marrow	NO
	Clonogenicity/Colony Formation	5, 25 µM	IEC-6	NO
		25 µM	Mouse bone marrow	NO
	In Vivo Survival	0.67 mg/mL (dw)	-	NO
Captopril	Viability	50, 100, 400 µM	IEC-6	NO
		400 µM	Mouse bone marrow	YES
	γ-H2AX	400 µM	IEC-6	YES
		400 µM	Mouse bone marrow	YES
	Clonogenicity/Colony Formation	400 µM	IEC-6	NO
		400 µM	Mouse bone marrow	YES
	In Vivo Survival	110 mg/kg (gav)	-	YES
PrC-210	Viability	0.5, 1, 2 mg/mL	IEC-6	YES
		0.5, 1, 2 mg/mL	Mouse bone marrow	YES
	γ-H2AX	1 mg/mL	IEC-6	YES
		1 mg/mL	Mouse bone marrow	YES
	Clonogenicity/Colony Formation	1 mg/mL	IEC-6	YES
		1 mg/mL	Mouse bone marrow	YES
	In Vivo Survival	900 mg/kg (ip)	-	YES
5-AED	Viability	0.5, 1 µM	IEC-6	YES
		0.5, 1 µM	Mouse bone marrow	NO
	γ-H2AX	0.5 µM	IEC-6	NO
		0.5 µM	Mouse bone marrow	YES
	Clonogenicity/Colony Formation	0.5 µM	IEC-6	NO
		0.5 µM	Mouse bone marrow	YES
	In Vivo Survival	30 mg/kg (sc)	-	YES
5-AET	Viability	0.5, 1, 2 µM	IEC-6	NO
		0.5, 1, 2 µM	Mouse bone marrow	NO
	γ-H2AX	0.5 µM	IEC-6	NO
		0.5 µM	Mouse bone marrow	NO
	Clonogenicity/Colony Formation	0.5 µM	IEC-6	NO
		0.5 µM	Mouse bone marrow	YES
	In Vivo Survival	30 mg/kg (sc)	-	NO

## Data Availability

The RNA sequencing data can be found at NCBI GEO, GSE295098. https://www.ncbi.nlm.nih.gov/geo/query/acc.cgi?acc=GSE295098.
